# New research progress on 18F-FDG PET/CT radiomics for EGFR mutation prediction in lung adenocarcinoma: a review

**DOI:** 10.3389/fonc.2023.1242392

**Published:** 2023-11-29

**Authors:** Xinyu Ge, Jianxiong Gao, Rong Niu, Yunmei Shi, Xiaoliang Shao, Yuetao Wang, Xiaonan Shao

**Affiliations:** ^1^ Department of Nuclear Medicine, the Third Affiliated Hospital of Soochow University, Changzhou, China; ^2^ Institute of Clinical Translation of Nuclear Medicine and Molecular Imaging, Soochow University, Changzhou, China; ^3^ Department of Nuclear Medicine, Changzhou Clinical Medical Center, Changzhou, China

**Keywords:** radiomics, machine learning, deep learning, EGFR, positron-emission tomography/computed tomography

## Abstract

Lung cancer, the most frequently diagnosed cancer worldwide, is the leading cause of cancer-associated deaths. In recent years, significant progress has been achieved in basic and clinical research concerning the epidermal growth factor receptor (EGFR), and the treatment of lung adenocarcinoma has also entered a new era of individualized, targeted therapies. However, the detection of lung adenocarcinoma is usually invasive. 18F-FDG PET/CT can be used as a noninvasive molecular imaging approach, and radiomics can acquire high-throughput data from standard images. These methods play an increasingly prominent role in diagnosing and treating cancers. Herein, we reviewed the progress in applying 18F-FDG PET/CT and radiomics in lung adenocarcinoma clinical research and how these data are analyzed via traditional statistics, machine learning, and deep learning to predict EGFR mutation status, all of which achieved satisfactory results. Traditional statistics extract features effectively, machine learning achieves higher accuracy with complex algorithms, and deep learning obtains significant results through end-to-end methods. Future research should combine these methods to achieve more accurate predictions, providing reliable evidence for the precision treatment of lung adenocarcinoma. At the same time, facing challenges such as data insufficiency and high algorithm complexity, future researchers must continuously explore and optimize to better apply to clinical practice.

## Introduction

Lung cancer is responsible for the highest cancer-associated mortality worldwide, among which lung adenocarcinoma, the most common pathological type, accounts for 40% of all lung cancers (1–3). Because of the insidious onset of symptoms at an early stage, advanced tumors have developed in nearly 80% of patients by the time of diagnosis, which results in a poor prognosis (4). Approximately 50% of Asian patients with adenocarcinoma harbor epidermal growth factor receptor (EGFR) mutations (5, 6). EGFR-mutant tumors exhibit an increased response rate to tyrosine kinase inhibitors (TKIs) versus EGFR wild-type tumors (7), while cisplatin-based chemotherapy yields more optimal outcomes in patients with EGFR wild-type lung cancer (8). Exon 19 deletion (19 del) and exon 21 L858R missense (21 L858R) are the two most frequent mutant isoforms of EGFR, which represent approximately 90% of all mutations (9). It has been shown that the 19 del mutation is more sensitive to TKIs than the 21 L858R mutation, leading to a longer median survival time (10). Afatinib or osimertinib is currently the first choice for treating patients with the 19 del mutation, whereas erlotinib plus bevacizumab therapy is recommended for patients with the 21 L858R mutation (11). Therefore, identifying EGFR mutation status and subtypes before treatment is crucial for offering accurate guidance regarding individualized patient treatment strategies.

Molecular detection of tumor tissues obtained by biopsy or surgical resection is the gold standard for identifying EGFR mutations (12). Because of the high risk of invasive procedures and the high heterogeneity of tumor tissues, pathological detection is not always feasible (13). Detection of circulating tumor DNA (ctDNA) has recently emerged as an alternative to EGFR mutation testing, but plasma ctDNA mutation detection can be easily affected by tumor burden and has a relatively high false-negative rate (14). Therefore, a noninvasive, rapid, and accurate method is urgently needed to identify EGFR mutations and subtypes.

Positron emission tomography (PET) imaging can visualize tissues at the biochemical level and detect potential neoplastic lesions earlier than computed tomography (CT) and magnetic resonance imaging (MRI) (15), while PET/CT can simultaneously provide metabolic and anatomical information regarding lesions. Recently, the relationship between the conventional metabolic parameters (such as maximum standardized uptake value (SUVmax)) of 18F-fluorodeoxyglucose (FDG) PET/CT and EGFR mutations were analyzed by many researchers, yet the results were controversial, probably because of the small sample size and complicated tumor microenvironment (16, 17).

First proposed by Lambin in 2012 (18), radiomics is continuously being perfected. Currently, radiomics refers to the interpretation of imaging data of regions of interest (ROIs), and thus, data with high-resolution spatial features are created using automated high-throughput feature extraction algorithms, which are further analyzed and interpreted by statistics and machine learning techniques, thereby supporting clinical decision-making (19). Over the past decade, 18F-FDG PET/CT radiomics has been applied to multiple aspects of cancer, such as the identification of benign and malignant solitary pulmonary nodules (20), lung cancer pathological typing and staging (21), prediction of gene mutation and molecular phenotypes (22), and evaluation of efficacy and prognosis (23). Amassed evidence substantiates the practicality and promising benefits of 18F-FDG PET/CT radiomics for prognosticating EGFR mutation classifications and subtypes (24). Radiomics methodology significantly mitigates patient discomfort and potential complications while delivering expedited diagnostic feedback and a comprehensive evaluation of tumor heterogeneity (25). Moreover, it serves as an invaluable tool for the dynamic monitoring of disease progression. Such advancements considerably improve patient experiences and promote efficient allocation of medical resources, thereby paving the way for developing tailored treatment regimens. This review aimed to summarize the research progress on 18F-FDG PET/CT radiomics for predicting EGFR mutation status and subtypes in lung adenocarcinoma.

### Literature search strategy

To gather information relevant to our topic, we conducted a comprehensive literature search in PubMed, using the following search terms: (((“Lung Neoplasms”[MeSH]) OR (Lung adenocarcinoma[Title/Abstract]) OR (Squamous cell carcinoma[Title/Abstract]) OR (Pulmonary Neoplasm[Title/Abstract]) OR (Lung Cancer[Title/Abstract]) OR (Pulmonary Cancer[Title/Abstract]) OR (NSCLC[Title/Abstract])) AND ((EGFR [Title/Abstract]) OR (epidermal growth factor receptor[Title/Abstract]) OR (targeted therapy[Title/Abstract]))) AND ((radiomics [Title/Abstract]) OR (deep learning[Title/Abstract]) OR (machine learning [Title/Abstract]) OR (transfer learning [Title/Abstract]) OR (CNN [Title/Abstract]) OR (Convolutional Neural Networks [Title/Abstract]) OR (features [Title/Abstract]) OR (radiogenomics [Title/Abstract]) OR (Artificial intelligence [Title/Abstract]))AND ((“ Positron Emission Tomography Computed Tomography”[MeSH]) OR (emission-computed tomography [Title/Abstract]) OR (PET [Title/Abstract])). Initially, we identified 83 articles. After a careful review of titles and abstracts, we selected 26 articles that met our topic.

### Procedures and classification of radiomics

**Figure 1:**
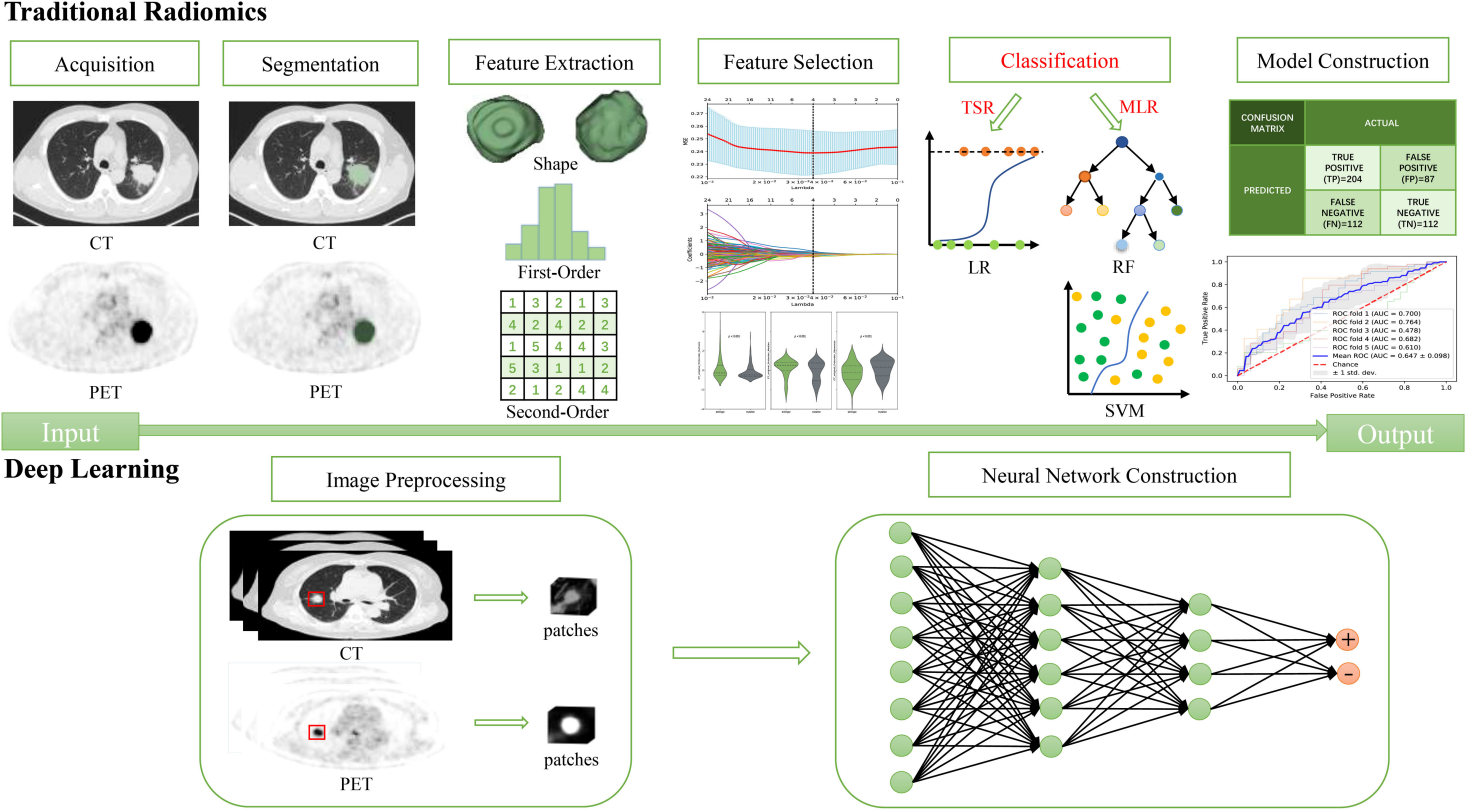
The differences in the procedures for the three radiomics.

Traditional radiomics (TR) procedures mainly consist of image acquisition, reconstruction, segmentation, feature extraction and filtering, model establishment, and performance evaluation (26). Multiple classifiers are used during the model establishment. Based on the different classifiers, the radiomics were classified in this study into traditional statistics-based radiomics (TSR), machine learning-based radiomics (MLR, specifically shallow learning), and deep learning (DL) to reflect iterations in omics technique. **Figure 1** illustrates the differences in the procedures for the three radiomics techniques.

TSR can identify the most valued features with non-zero coefficients by extracting TR features (e.g., shape-based features, histogram features, and texture features) and applying mathematical-statistical methods integrated with algorithms such as the least absolute shrinkage and selection operator (LASSO) (27). A radiomics score (Rad-score) is then calculated and established for each lesion with a linear combination of the selected features correspondingly weighted by their coefficients. A classical logistic regression (LR) approach is then adopted as the classifier to build radiomics models. This method is the earliest, simplest, and most interpretable omics technique.

MLR is the most mature and mainstream approach that establishes classification or prediction models using machine learning algorithms after feature extraction and optimal radiomics feature screening. Common machine learning classifiers encompass random forest (RF), support vector machine (SVM), decision tree (DT), Bayesian network (BN), and k-nearest neighbor (KNN) algorithms (28). After MLR, there is a unique method named deep learning-based radiomics (DLR) that can extract DL features using artificial neural networks (ANNs), such as convolutional neural networks (CNNs), and then construct models using machine learning algorithms (29, 30).

DL, different from the TR represented by TSR and MLR, relies on multilayer nonlinear neural networks and bypasses the cumbersome feature extraction and selection procedures to automatically learn features from images, by which prediction models are established according to the “end-to-end” workflow without any human intervention (31).

### Prediction of EGFR mutations by the TSR model

Before the emergence of radiomics, the clinical characteristics of patients with lung adenocarcinoma were frequently applied to predict EGFR mutation status, and multiple clinical characteristics (e.g., female, non-smoker, and adenocarcinoma histology) were related to EGFR mutations (32). It has also been shown that several CT features, such as maximum tumor diameter, tumor location, density, ground-glass opacity, pleural traction, and air bronchogram, denote EGFR mutation status in lung adenocarcinoma (33). A recent study has illustrated that tumors with reduced long diameters have a slightly higher risk of EGFR mutations though tumors with ground-glass opacity have markedly risk of EGFR mutations (34). Yip et al. (35, 36) demonstrated that PET radiomic features have great potential to predict EGFR mutation status by quantifying the tumor metabolic phenotype. However, the radiomics features usually can only reflect information regarding the image and cannot comprehensively reflect the patient’s condition. To increase the model’s accuracy in identifying EGFR mutation status and subtypes, several researchers incorporated clinical information, including CT features, into 18F-FDG PET/CT radiomics models (34).

In recent years, it has been revealed that the diagnostic performance and goodness-of-fit of models utilizing clinical features integrated with TR features are more optimal, with greater clinical benefit in predicting EGFR mutations. Eight studies on TSR are listed in **Table 1**, six of which used combined models of radiomics and clinical features (22, 34, 37, 40–42) and achieved superior performance as compared to single radiomics models and single clinical feature models, with the area under the curve (AUC) values of the test set results ranging from 0.81 to 0.87. These data demonstrate the effectiveness of this integrated imaging tool in predicting EGFR mutations.

**Table 1:** Studies on applying 18F-FDG PET/CT TSR in predicting EGFR mutations in patients with lung adenocarcinoma.

Author	Year	Samples	Radiomics features	Training set	Testing set
Li et al. (22)	2022	179 (EGFR+: 105, EGFR-: 74; 19 del: 46, 21 L858R: 53)	2 PET radiomics features; 4 CT radiomics features	mutant/wild model PET/CT + clinical: AUC= 0.882; 19 del/21 L858R model: PET/CT: AUC = 0.708	mutant/wild model PET/CT + clinical: AUC= 0.837; 19 del/21 L858R model: PET/CT: AUC = 0.652
Zhao et al. (37)	2022	88	6 PET radiomics features; 6 CT radiomics features	PET/CT + clinical: AUC = 0.864	–
Nair et al. (38)	2021	50 (EGFR+: 21, EGFR-: 29; 19 del: 11, 21 mut: 10)	14 PET/CT radiomics features	mutant/wild model: PET/CT: AUC = 0.8713 ± 0.05 exon19/21 model: PET/CT: AUC = 0.860 ± 0.07	–
Yang et al. (39)	2021	114 (EGFR+:51, EGFR-:63)	7 PET/CT radiomics features	PET/CT: AUC = 0.866	–
Chang et al. (34)	2021	583 (EGFR+: 295, EGFR-: 288)	30 PET/CT radiomics features; 12 PET features; 15 CT features	PET/CT: AUC= 0.76; PET/CT + clinical: AUC= 0.84	PET/CT: AUC= 0.75; PET/CT + clinical: AUC= 0.81
Zhang et al. (40)	2020	173 (EGFR+: 71, EGFR-: 102; 19 del: 29, 21 L858R: 38)	2 PET radiomics features; 4 CT radiomics features	PET/CT: AUC= 0.868, PET/CT + clinical: AUC= 0.866	mutant/wild model PET/CT: AUC= 0.769, PET/CT + clinical: AUC= 0.827, 19 del/21 L858R model: AUC = 0.661;
Zhang et al. (41)	2020	248 (EGFR+: 133, EGFR-: 115)	5 PET radiomics features; 5 CT radiomics features	PET/CT: AUC= 0.79; PET/CT + clinical: AUC= 0.86	PET/CT: AUC= 0.85; PET/CT + clinical: AUC = 0.87
Li et al. (42)	2019	115 (EGFR+: 64, EGFR-: 51)	2 PET radiomic features; 4 CT radiomic features	PET/CT: AUC= 0.805; PET/CT + clinical: AUC= 0.822	–

AUC, area under the curve; EGFR, epidermal growth factor receptor; 19 del, 19 deletion; 21 L858R, 21 L858R missense; PET, positron emission tomography; CT, computed tomography.

Regarding discriminating the main subtypes of EGFR mutations, studies have shown a relatively low predictive accuracy of 18F-FDG PET/CT radiomics to distinguish the 19 del mutation from 21 L858R. Li et al. (22) reported AUC values of 0.708 and 0.652 in predicting the 19 del mutation and 21 L858R mutation in the training and testing sets, respectively. In the study of Zhang et al. (40), after clinical information was integrated into the predictive model, only one PET feature could discriminate between the 19 del mutation and 21 L858R mutation, showing low predictive ability (AUC = 0.661). Additionally, Nair et al. (38) suggested that PET/CT features were superior (AUC = 0.86) for discriminating mutations in exons 19 and 21 compared to CT features alone. However, this study had a smaller sample size of only 21 patients and lacked an independent test set. It was considered that the poor predictive performance for EGFR mutation subtypes was mainly attributed to overfitting of the trained model caused by inherent technical limitations of TSR, such as but not limited to the possible loss of essential information with the use of single LASSO regression due to high dimensionality of the radiomics features (43).

Radiomics has rapidly developed as an emerging field, but TSR continues to be used mainly due to its advantages of good interpretability and easy application in clinical practice. TSR can effectively transform the data into scores, usually in the form of a nomogram (23, 34, 39), which allows intuitive calculation and easier understanding and analysis of the associations and differences between the data. However, its limitation is the low predictive efficacy of the traditional logistic regression model relative to most machine learning classifiers, which is also why the use of MLR is becoming increasingly common.

### Prediction of EGFR mutations by the MLR model

Most machine learning algorithms aim to develop an optimal model to solve one problem. The selection of machine learning classifiers requires comprehensive consideration of multiple factors such as data characteristics, model performance, computational resources, and usage, and experiments are necessary to determine the optimal classifier (44, 45). Studies on EGFR mutation identification in lung cancer based on different machine learning classifiers are presented in **Table 2**.

**Table 2:** Studies on applying 18F-FDG PET/CT MLR in predicting EGFR mutations in patients with lung adenocarcinoma.

Author	Year	Samples	Model	Training set	Testing set
Gao et al. (46)	2023	515 (EGFR+:313, EGFR-:202)	**RF,** LR, SVM	AUC=0.760	AUC=0.730
Zhang et al. (47)	2023	115 (EGFR+:64, EGFR-: 51)	**LR**, RF, SVM, Adaboost	AUC=0.843	–
Zuo et al. (48)	2023	767 (EGFR+:450, EGFR-: 317)	**LGBM,** XGB, RF,LR	–	mutant/wild model: AUC=0.80; 19 del/21 L858R model: AUC=0.76
Yang et al. (49)	2022	313 (EGFR+:181, EGFR-:132)	**SVM**, DT, RF	mutant/wild model: AUC=0.881; 19 del/21 L858R model: AUC_19 del_=0.849; AUC_21 L858R_=0.851	mutant/wild model: AUC=0.926; 19 del/21 L858R model: AUC_19 del_=0.859; AUC_21 L858R_=0.805
Agüloğlu et al. (50)	2022	159 (EGFR+:59, EGFR-:100)	RF, **NB**, KNN, LR, SVM, DT	AUC=0.751	AUC=0.797
Shiri et al. (51)	2022	136	RF	AUC=0.92–0.94	–
Ruan et al. (52)	2022	100 (EGFR+:46, EGFR-: 54)	LR, **SVM**	AUC=0.746	AUC=0.741
Huang et al. (30)	2022	138 (EGFR+:64, EGFR-: 74)	CNN	AUC=0.91	AUC=0.85
Wang et al. (43)	2021	238 (EGFR+: 126, EGFR-: 112)	KNN, SVM, **Adaboost**	19 del/21 L858R model: AUC=0.87	–
Shiri et al. (53)	2020	300	SVM, KNN, DT, QDA, MLP, SGD, LR, NB, GNB, **RF,** AB, BAG	AUC=0.82	–
Yang et al. (54)	2020	174 (EGFR+:109, EGFR-:65)	RF	mutant/wild model: AUC=0.77; 19 del/21 L858R model: AUC=0.82	mutant/wild model: AUC=0.71; 19 del/21 L858R model: AUC=0.73
Liu et al. (55)	2020	148 (EGFR+: 75, EGFR-: 73)	XGB	mutant/wild model: AUC=0.93; 19 del/21 L858R model: AUC_19 del_=0.91; AUC_21 L858R_=1.0	mutant/wild model: AUC=0.87; 19 del/21 L858R model: AUC_19 del_=0.77; AUC_21 L858R_=0.92
Koyasu et al. (56)	2020	138 (EGFR+:38, EGFR-:100)	RF, **XGB**	AUC=0.659	–
Jiang et al. (57)	2019	80 (EGFR+:30, EGFR-:50)	SVM	AUC=0.953	–

KNN, K-nearest neighbor; DT, decision tree; QDA, quadratic discriminant analysis; MLP, multilayer perceptron; SGD, stochastic gradient descent; LR, logistic regression; NB, naive Bayes; GNB, Gaussian naive Bayes; RF, random forest; AB, adaptive boosting; BAG, bagging; SVM, support vector machine; XGBoost, eXtreme gradient boosting; Adaboost, adaptive boosting; LGBM, light gradient boosting machine classifier; CNN, convolutional neural network. Bold indicates the best performance model.

LR is a simple and easily implementable algorithm for binary classification problems. Zhang et al. (47) found that the fusion of PET and CT image features did not significantly enhance the classifier’s performance compared to using only PET image features. Furthermore, simple and efficient classifiers tend to be more advantageous in situations with limited data. Therefore, they utilized a small amount of single-mode PET images, employed various methods to extract features and perform dimensionality reduction, and then input the most significant features into the LR model for classification. This approach ultimately yielded the highest AUC value of 0.843. This method used less data than previous research but achieved better results.

The RF algorithm, one of the most classical machine learning algorithms, is a decision tree-based ensemble learning algorithm that integrates the results obtained by several weak classifiers to improve the model performance. Gao et al. (46) found that compared with LR and SVM, the RF performed best among the three radiomics models of CT, PET, and PET/CT (testing sets AUC: 0.726, 0.678, and 0.704). Yang et al. (54) adopted the RF algorithm to develop a predictive model for EGFR mutations and determined its AUC values of 0.77 and 0.71 in the training set and testing set, respectively.

XGBoost algorithm, derived from the RF algorithm, is a gradient-boosting decision tree-based model algorithm with a strong modeling effect and ultrafast computational speed. It increases computational efficiency with its parallel computation and cache optimization. Also, there is greater stability associated with the XGBoost algorithm due to its application of a regularization technique in the training process. Liu et al. (55) employed XGBoost to predict EGFR mutations and found that the 18F-FDG PET/CT radiomics model achieved satisfactory capability for identifying EGFR mutation status (AUC = 0.87). It was reported by Koyasu et al. (56) that a stronger performance was observed with a radiomics model employing XGBoost and multiple types of imaging features as compared to RF, and this model showed the most optimal performance in the classification of EGFR mutations (AUC = 0.659).

SVM, another frequently used supervised learning algorithm, accomplishes satisfactory nonlinear data-fitting because SVM is a very commonly used strong learner with excellent performance in finding the optimal solution in high-dimensional space using a kernel function. After a comparison, Ruan et al. (52) revealed that the SVM model outperformed the LR model in EGFR mutation identification, and particularly, the SVM model based on the radial basis kernel function exhibited the most optimal performance in the testing set (AUC = 0.741). Yang et al. (49) retained the radiomics features and clinical factors to establish integrated models. The SVM model exhibited a stronger performance than the RF and DT models, yielding an AUC value of 0.926 in the testing set. Jiang et al. (57) found that combining qualitative and quantitative features outperformed qualitative or quantitative features alone, and the SVM model achieved excellent performance with a high AUC of 0.953.

The naive Bayes (NB) algorithm is a supervised learning algorithm based on Bayes’ theorem that is computationally efficient, rapid, and simple to operate. Agüloğlu et al. (50) found that the NB machine learning algorithm established using the GLZLM_GLNU feature and clinical data contributed to the most successful prediction of EGFR mutations (AUC = 0.751). Shiri et al. (53) developed models using multimodal PET/CT image features and trained them using multiple machine learning methods for EGFR mutation prediction, achieving an AUC value of 0.82, which was increased to 0.94 by ComBat harmonization (51). Further optimization, such as data correction, may improve the model’s performance in case of unsatisfactory results.

Studies have demonstrated the potential of 18F-FDG PET/CT radiomics to discriminate EGFR subtypes. Liu et al. (55) obtained two sets of prognostic radiomics features for specific EGFR mutation subtypes and found that their XGBoost classifier for the 19 del and 21 L858R mutations yielded a prediction accuracy of 0.77 and 0.92 with respect to AUC values, respectively. The study of Yang et al. (54) utilized an RF classifier, and the resultant AUC values of the models for discriminating 19/21 site mutations were 0.82 and 0.73 in the training test and testing set, respectively.

Wang et al. (43) developed a PET/CT radiomics model using an Adaboost classifier and reported its predictive value for EGFR mutation subtypes (AUC = 0.86). Adaboost is also an ensemble learning algorithm that can train a weak classifier through multiple iterations, improving overall accuracy. Zuo et al. (48) discovered that the XGBoost classifier combined with SVM feature selection method achieved the best performance in predicting EGFR subtypes (AUC reached 0.76, 0.63, and 0.61 in the internal test cohort and two external test cohorts, respectively). Also, Yang et al. (49) revealed that the SVM model was superior to the RF model and the DT model, achieving AUC values of 0.805 and 0.859 for predicting the 19 del and 21 L858R mutations in the testing set, respectively. Based on the results of subtype prediction, the predictive performance for the exon 21 mutation was more optimal than that for the exon 19 mutation, and the overall results for MLR were also superior to those of TSR.

Particularly, with the continuous development of AI technologies, DL, represented by CNNs, has shown satisfactory performance. Radiomics gradually relies on DL techniques to address challenges and limitations in routine radiomics workflows, including automated detection and segmentation procedures and harmonizing images by synthetic generation (58). To reduce or balance the high cost of data acquisition, several researchers have proposed DLR to integrate the feature outputs from the DL networks with classical machine learning classifiers. Huang et al. (30) constructed a DLR model to predict EGFR mutations using three-dimensional (3D) CNN, and they found that this DLR model was superior to TR (AUC: 0.79 *vs*. 0.68). DLR, as a transition between TR and DL, extracts and classifies advanced features and image data using multilayer neural networks, improving the accuracy of data analysis. There are few studies on DLR in this field, and additional studies may be required in the future to continually explore and validate the feasibility and performance of this proposal.

EGFR prediction models established using machine learning algorithms can identify EGFR mutation status and subtypes, displaying satisfactory discrimination, prediction accuracy, correction performance, and gain values. There have been more reports on MLR in the last few years, and the machine learning algorithms applied in this method have also been more mature than those of TSR. In the field of machine learning, there is no single algorithm that performs best in all situations. The performance of an algorithm depends on various factors, such as the data’s characteristics, the problem’s complexity, and the algorithm’s parameter tuning. For a specific problem, it is necessary to experimentally compare different algorithms’ performance to determine which is the most suitable. Generally, MLR selects the optimal combination to achieve stronger results using multiple feature screening and multiple classifiers. Based on our summary, RF and SVM are the classifiers that usually perform best in prediction efficacy. In radiomics, fusion using the stacking algorithm is an effective approach (59) because it incorporates the obtained multiple results of classifiers, where the prediction results of various models are selected as a new input and then trained into one model to improve the classification performance. No studies concerning this approach in MLR have been performed, but this approach may become more common with advanced technologies and data accumulation.

### Prediction of EGFR mutations by the DL model

Radiomics features can be classified into two types: the first refers to predefined or manually extracted features established by an image processing expert, also known as traditional features; the second refers to deep features, and several DL algorithms assign a task in their extraction layer to self-design and select features without any human intervention (31, 58). TSR and MLR extract similar and traditional features, which mainly differ concerning different classifiers, while DLR and DL extract deep features. Several studies have suggested that deep features are superior to traditional features (60, 61).

Recently, DL has shown great potential in improving feature engineering in medical imaging and classification and prediction accuracy (62). Several investigators are now focusing on applying DL models in predicting EGFR mutation status, which has shown promising performance. Xiao et al. (63) proposed a deep learning framework based on the EfficientNet-V2 model. First, 32 2D views are extracted from each 3D cube of lung nodules. Then, deep features are extracted from these 32 views to predict whether EGFR is mutated. The results show that this deep learning model outperforms the radiomics model, with AUCs of 83.64% and 82.41%, respectively, effectively predicting EGFR mutations.

Using the robust deep convolutional neural network structure, the squeeze-and-excitation residual network (SE-ResNet) module, Yin et al. (64) developed two DL models (SE_CT_ and SE_PET_) for EGFR mutation identification in CT and PET images, respectively. They also integrated the results of SE_CT_ and SE_PET_ using stacked generalization and increased the AUC value to 0.84, which was significantly higher than that of either SE_CT_ or SE_PET_.

In another study, Chen et al. (65) applied the Stack DL model to effectively integrate anatomical bioimaging data from PET/CT images with clinical data, which displayed a strong predictive ability for EGFR mutations (AUC = 0.85 ± 0.09) and was superior to the ResNet PET/CT model (AUC = 0.81 ± 0.07) and the radiomics model (AUC = 0.60 ± 0.06). The most optimal features were constructed from the image data rather than selected from a predefined and limited set of feature candidates. With sufficient training data, CNN will outperform the feature selection scheme.

Mu et al. (66) developed an 18F-FDG PET/CT-based DL model using the 2D small-residual-convolutional-network (SResCNN), which accurately classified EGFR mutation status. Also, after constructing an integrated model that included EGFR-DLS (DL score) and histological and clinical features (smoking), the integrated model showed superior performance to the DL or clinical model (AUC = 0.88, 0.83, and 0.78, respectively). Overall, the multitask artificial intelligence system incorporating DL clinical features achieved relatively satisfactory results in predicting EGFR status. This finding illustrates that incorporating multiple data can partly enhance the model’s predictive accuracy.

At present, the use of 18F-FDG PET/CT DL models for EGFR mutation status prediction has been scarcely studied, while its use in EGFR mutation subtype identification has not been discussed. Although three retrieved articles (**Table 3**) demonstrated clear discrimination of EGFR mutations, all of them acquired section samples from 3D input images and thus obtained sufficient data to train 2D-CNN *de novo*, which would result in the loss of rich 3D anatomical information (67). In addition, DL-dependent methods in radiomics face some problems and need to address new challenges, one of which is the limited size of the data set. Generally, DL training requires less human input than traditional ML algorithms, but effective DL often involves a mass of training data.

**Table 3:** Studies on applying 18F-FDG PET/CT DL in predicting EGFR mutations in patients with lung adenocarcinoma.

Author	Year	Samples	Model	Training set	Testing set
Xiao et al. (63)	2023	150 (EGFR+:57, EGFR-: 93)	EfficientNet-V2	–	AUC%=83.64 ± 2.41
Chen et al. (65)	2022	147 (EGFR+:37, EGFR-: 110)	ResNet	StackPET-CT+ clinical: AUC=0.85 ± 0.09	–
Yin et al. (64)	2021	301 (EGFR+:153, EGFR-: 148)	SE-ResNet	StackPET-CT: AUC=0.86	StackPET-CT: AUC=0.84
Mu et al. (66)	2020	681 (EGFR+:312, EGFR-:369)	SResCNN	DLS+ clinical: AUC=0.88	DLS+ clinical: AUC=0.88

ResNet, residual network; SE-ResNet, squeeze-and-excitation residual network; SResCNN, small-residual-convolutional-network; DLS, deep learning score.

In medical imaging, obtaining such many training samples is generally difficult. This limitation can theoretically be addressed by the similarity of visual features in the problem domain using the transfer learning (TL) strategy (44). Indeed, although TL is effective in DL, its application is limited because of the lack of new pre-trained models for medical imaging (68, 69). In recent years, scholars have also proposed leveraging unlabeled data (i.e., semi-supervised learning) to address the issue of small sample sizes, including techniques like pseudo-labeling and Generative Adversarial Networks (GANs) (70, 71). The potential value of these innovative approaches in predicting EGFR mutations remains an area ripe for further exploration and research in the future (72). Second, considerable computational resources are usually required to train DL models, including central processing units (CPUs), graphics processing units (GPUs), and memory. If computational resources are limited, TR may be a more optimal choice. The lack of interpretability of DL network-based models is an additional issue that remains incompletely addressed, and this algorithm is usually called the “black box” (73). The high performance of deep neural networks is achieved at the cost of high complexity and many parameters. Although one can, in principle, follow every processing step, one cannot understand the internal decision-making process because many parameters make it difficult to obtain meaningful explanations of model behaviors in this way.

There is insufficient ability for traditional machine learning techniques to process raw data, while DL models are well-suited for model training with large-scale data in the current big data era, and their high accuracy is due to their multilayer structure that can capture complex relations in the data and process high-dimensional and nonlinear data (31). DL models can learn more representative features than TR models, which is critical for stronger analytical performance. TR methods require rigorous procedures, such as detection, segmentation, feature extraction, and selection, which are cumbersome and time-consuming (74). DL can greatly improve efficiency through its “end-to-end” learning mode. Furthermore, radiomics features can be easily affected by artificial segmentation and scanning parameters, while DL models can adaptively learn features based on the data, thus effectively improving the model’s robustness and generalization ability (29). Currently, several visualization methods have been developed to interpret the decision-making process of DL models. The most common method is using gradient-weighted class activation mapping (Grad-CAM) to generate “heatmaps” for input images, showing the influence weights of different parts of the image on the classification results, thereby explaining the learning process (75). In summary, deep learning and machine learning are advantageous in different applications. The most suitable approach in practice often depends on the specific requirements and constraints of the task. In the era of precision medicine, MLR will combine with DL to achieve automated imaging analysis and diagnosis, thereby improving diagnostic accuracy and efficiency. The advantages and disadvantages of the three types of radiomics are listed in **Table 4**.

**Table 4:** Advantages and disadvantages of different types of radiomics.

Radiomics type	Advantages	Disadvantages
**TSR**	1. Methods are based on explicit assumptions and statistical models, and the results can be mathematically demonstrated. 2. The data are easy to interpret and understand and can provide meaningful biological results. 3. The requirement for data is low, and a mass of data is not required.	1. Provides a poor fit to complex data relations. 2. The analytical performance for high-dimensional data is limited, and complex nonlinear relations are poorly processed. 3. Extensive feature engineering may be required, which requires researchers to have extensive knowledge of biology.
**MLR**	1. Accuracy is high with satisfactory applicability. 2. The model is more interpretable and comprehensible. 3. High-dimensional data and nonlinear relationships can be processed.	1. Overfitting is easy in insufficient training data or noise. 2. Only labeled data can be processed. 3. Adjusting parameters is difficult and requires the strong technical skills of researchers.
**DL**	1. High performance for large-scale and high-dimensional data processing. 2. Data features can be automatically learned with a satisfactory fit to complex data relationships. 3. Unstructured data processing performance is satisfactory.	1. Poor interpretability with difficulty in understanding the results. 2. Requires substantial computational resources and data. 3. There are strict requirements for training data, and poor data quality may affect the results.

TSR, traditional statistics-based radiomics; MLR, machine learning-based radiomics; DL, deep learning.

### Limitations and prospects

Identifying EGFR mutation status in lung adenocarcinoma using 18F-FDG PET/CT radiomics is promising, but limitations remain. First, the scanner and scanning parameters have not yet been standardized, and there has been no consensus on delineating lesional ROIs, which will partly affect the objectivity and reproducibility of experiments. As a standardization guideline, the Image Biomarker Standardization Initiative (IBSI) has proposed uniform standards for image quality, feature extraction, and preprocessing modalities to ensure the robustness and reproducibility of experiments (76, 77). Also, data sharing is a huge obstacle for researchers. Sharing training parameters instead of data is a simple solution (78, 79).

Second, similar to multidisciplinary teams in the clinical treatment of lung cancer, integrating different features is also conducive to constructing prediction models (80), yielding the concept of multi-omics (81). Combining knowledge from different fields and multiple disciplines, such as imaging, pathology, statistics, and clinical data, and the convergence of old and new techniques is a worthwhile objective for the future.

Additionally, the current studies on radiomics are mostly single-center, small-scale retrospective studies, and prospective studies and external independent validation are scarce, which may lead to the overfitting of data and insufficient generalization ability of models. Thus, multi-center collaboration will be necessary to build large databases to increase models’ accuracy. Furthermore, limited specificity and sensitivity are the inherent bottlenecks in the application of the tracer 18F-FDG in predicting lung cancer driver genes, and new imaging methods and new molecular probes such as 18F-MPG are required to improve the model performance (66, 82).

Furthermore, there are currently few studies on the application of PET/CT in identifying EGFR mutation subtypes, and two binary classification models are adopted in most studies on EGFR mutation subtypes. The ternary classification model can be directly applied in the future to obtain richer information that better describes the true situation of the samples, thus improving the prediction accuracy of models. However, the training and evaluation of ternary classification models are more complicated, requiring additional data and a longer time.

Moreover, habitat imaging is an important component of radiomics and a hot topic in cancer quantitative imaging. This generally refers to the analysis of different sub-regions within a tumor. These sub-regions are referred to as “habitats”, each of which may represent different biological characteristics and pathophysiological processes within the tumor (83). By analyzing these habitats, we can better understand the heterogeneity of the cancer, thereby facilitating the development of personalized treatment plans.

Lastly, although these models have clear advantages, their widespread clinical application still faces multiple challenges, such as data heterogeneity, interpretability of the models, difficulties in clinical integration, the need for external validation, regulatory barriers, economic considerations, and ethical and privacy concerns (84). In the future, we can further explore these models’ practical application potential and clinical translation ability through prospective clinical research.

## Conclusions

Radiomics is a well-established research method, forming a complete theoretical system and research process, but AI-based radiomics in medicine remains in the initial stage and is not widely utilized. With the advancement of machine learning methods and the continuous development of DL technologies, identifying EGFR mutation status by radiomics in lung adenocarcinoma will continue to be an area of intense clinical medicine research, providing more precise imaging diagnosis and individualized therapeutic guidance for patients.

## Author contributions

XG is responsible for literature search and manuscript writing; JG, RN, XLS, YS, and YW are accountable for content planning and editing. XNS is responsible for proposing the topic and revising the content. All authors have read the final manuscript and approved the version to be published. All authors contributed to the article and approved the submitted version.

## Glossary

**Table:**

19 del	exon 19 deletion
21 L858R	exon 21 L858R missense
3D	three-dimensional
AB/Adaboost	adaptive boosting
ANN	artificial neural networks
AUC	area under the curve
BAG	bagging
BN	Bayesian network
ctDNA	circulating tumor DNA
CNN	convolutional neural network
CPU	central processing unit
DL	deep learning
DLR	deep learning-based radiomics
DLS	DL score
DT	decision tree
EGFR	epidermal growth factor receptor
FDG	fluorodeoxyglucose
GANs	Generative Adversarial Networks
GNB	Gaussian naive Bayes
GPU	graphics processing unit
Grad-CAM	gradient-weighted class activation mapping
IBSI	image biomarker standardization initiative
KNN	k-nearest neighbor
LASSO	least absolute shrinkage and selection operator
LR	logistic regression
MLP	multilayer perceptron
MLR	machine learning-based radiomics
MRI	magnetic resonance imaging
NB	naive Bayes
PET	positron emission tomography
QDA	quadratic discriminant analysis
Rad-score	radiomics score
ResNet	Residual network
RF	random forest
ROI	region of interest
SE-ResNet	Squeeze-and-excitation residual network
SGD	stochastic gradient descent
SResCNN	small-residual-convolutional-network
SUVmax	maximum standardized uptake value
SVM	support vector machine
TKI	tyrosine kinase inhibitors
TR	traditional radiomics
TSR	traditional statistics-based radiomics
XGBoost	eXtreme gradient boosting
